# Epizootic Haemorrhagic Disease Virus Serotype 8 in Tunisia, 2021

**DOI:** 10.3390/v15010016

**Published:** 2022-12-21

**Authors:** Soufien Sghaier, Corinne Sailleau, Maurilia Marcacci, Sarah Thabet, Valentina Curini, Thameur Ben Hassine, Liana Teodori, Ottavio Portanti, Salah Hammami, Lucija Jurisic, Massimo Spedicato, Lydie Postic, Ines Gazani, Raja Ben Osman, Stephan Zientara, Emmanuel Bréard, Paolo Calistri, Jürgen A. Richt, Edward C. Holmes, Giovanni Savini, Francesca Di Giallonardo, Alessio Lorusso

**Affiliations:** 1 Institut de la Recherche Vétérinaire de Tunisie, Tunis 1006, Tunisia; 2 UMR VIROLOGIE, INRAE, École Nationale Vétérinaire d’Alfort, ANSES Laboratoire de Santé Animale, Université Paris-Est, 94700 Maisons-Alfort, France; 3 Istituto Zooprofilattico Sperimentale dell’Abruzzo e del Molise, 64100 Teramo, Italy; 4 Direction Générale des Services Vétérinaires, Commissariat Régional au Développement Agricole de Nabeul, Nabeul 1082, Tunisia; 5 Service de Microbiologie, Immunologie et Pathologie Générale, École Nationale de Médecine Vétérinaire de Sidi Thabet, IRESA, Universitè de la Manouba, Winnipeg 2010, Tunisia; 6 Facoltà di Medicina Veterinaria, Università degli Studi di Teramo, 64100 Piano D’Accio-Teramo, Italy; 7 CRDA Ministère d’Agriculture, Avenue Habib Bourguiba, Kasserine 1200, Tunisia; 8 National Drug Control Laboratory, Vaccine Control Unit, Tunis 1002, Tunisia; 9 Department of Diagnostic Medicine/Pathobiology, College of Veterinary Medicine, Kansas State University, Manhattan, KS 66506, USA; 10 Sydney Institute for Infectious Diseases, School of Medical Sciences, The University of Sydney, Sydney 2006, Australia; 11 The Kirby Institute, The University of New South Wales (UNSW), Sydney 2052, Australia

**Keywords:** EHDV, Tunisia, virus characterization, EHDV serotype 8, circulation

## Abstract

Epizootic haemorrhagic disease (EHD) is a *Culicoides*-borne viral disease caused by the epizootic haemorrhagic disease virus (EHDV) associated with clinical manifestations in domestic and wild ruminants, primarily white-tailed deer (*Odocoileus virginianus*) and cattle (*Bos taurus*). In late September 2021, EHDV was reported in cattle farms in central/western Tunisia. It rapidly spread throughout the country with more than 200 confirmed outbreaks. We applied a combination of classical and molecular techniques to characterize the causative virus as a member of the serotype EHDV-8. This is the first evidence of EHDV- 8 circulation since 1982 when the prototype EHDV-8 strain was isolated in Australia. This work highlights the urgent need for vaccines for a range of EHDV serotypes.

## 1. Introduction

The Epizootic haemorrhagic disease (EHD) is a vector-borne disease of wild and domestic ruminants caused by the EHD virus (EHDV). This virus belongs to the genus *Orbivirus* of the family *Sedoreoviridae* (ICTV, https://ictv.global/report/chapter/sedoreoviridae/sedoreoviridae/orbivirus accessed on 8 November 2022) and is closely related to the Bluetongue virus (BTV) [1]. Both viruses are transmitted by *Culicoides* biting midges (Diptera: Ceratopogonidae). EHD is a common disease in wild ruminants, particularly among white-tailed deer (*Odocoileus virginianus*) in North America, while mule deer (*Odocoileus hemionus*) and pronghorn (*Antilocapra americana*) are affected to a lesser extent. EHDV infection in deer often results in high levels of mortality associated with high fever, lethargy, oedema, ulcerations of the dental pad and oral mucosa, hemorrhaging of the heart, lungs, major blood vessels and other tissues. Historically, less severe or asymptomatic infections are observed in cattle (*Bos taurus*), which are considered to be the reservoir host for the virus [2,3,4,5]. EHDV has been detected globally across tropical and temperate regions of the Americas, Asia, Africa, Australia, and the Middle East. In contrast to BTV, however, EHDV has never been described in Europe.

EHDV genome comprises 10 linear segments of double-strand RNA, coding seven structural (VP1–VP7) and four non-structural (NS1–NS4) proteins [6]. The outer capsid protein—VP2—is the primary determinant of serotype specificity. Seven distinct serotypes are recognized by phylogenetic clustering and genetic distances (1, 2, 4–8). However, the relationship between the individual serotypes has not been fully determined and serotype classification is challenging. Reclassification led to the previous serotype 3 now being recognized to be part of EHDV serotype 1, and EHDV-318 (also referred to as EHDV-9) now classified as EHDV-6 [7]. In addition, the highly virulent Ibaraki virus, first identified in cattle in Japan in 1959, is now considered a member of EHDV-2 [8]. A number of putative novel serotypes have also been described [9,10]. The only commercially available vaccines are a monovalent live-attenuated vaccine and a bivalent (EHDV-2/bovine ephemeral fever virus) inactivated vaccine produced in Japan for the control of the Ibaraki strain. In addition, EHDV is controlled in North America by using autogenous vaccines [11].

In the past two decades, there has been a gradual increase in the number of EHD outbreaks in cattle. The circulation of different EHDV serotypes have been documented in Ecuador (EHDV-1) [12] the island of Mayotte (EHDV-6, and 7) [13], Trinidad (EHDV-6), French Guiana (EHDV-1 and 6) [14], Egypt (EHDV-1) [15], Israel (EHDV-1, -6 and -7) [16,17,18], Maghreb (EHDV-6) [19,20], Arabian Peninsula and African countries [4], and China (EHDV-7) [21]. In these outbreaks, the cattle showed BTV-like clinical signs including fever, anorexia, facial oedema, dysphagia, ulcerative and necrotic lesions of the mouth, reduction in rumination, respiratory distress, hyperemia of teats and udder, difficulty swallowing, lameness and drop in milk production. Death, abortions, and stillbirths were also reported in some cases.

In Tunisia, the first EHDV outbreak was described in 2006, causing high mortality and morbidity, as well as large economic losses for different cattle farms. Based on sequencing analyses of segment 2 (Seg-2), the EHDV strains from Tunisia were classified as serotype 6 (EHDV-6) and were closely related to other EHDV-6 strains circulating in the Mediterranean basin [19]. In late September 2021, EHDV was reported in cattle farms in central/western Tunisia and then spread rapidly across the Northern and Eastern regions between October and November, with more than 200 confirmed outbreaks. Herein, we used a combination of classical and molecular techniques to characterize the causative virus of the 2021 outbreak.

## 2. Materials and Methods

### 2.1. The Ethical Statement

The study did not involve any animal experimentation. Blood and serum samples were collected by the Tunisian Veterinary Services within the context of outbreaks’ investigation, following standard procedures, such that no ethical approval was required.

### 2.2. Specimen Collection

**Figure 1:**
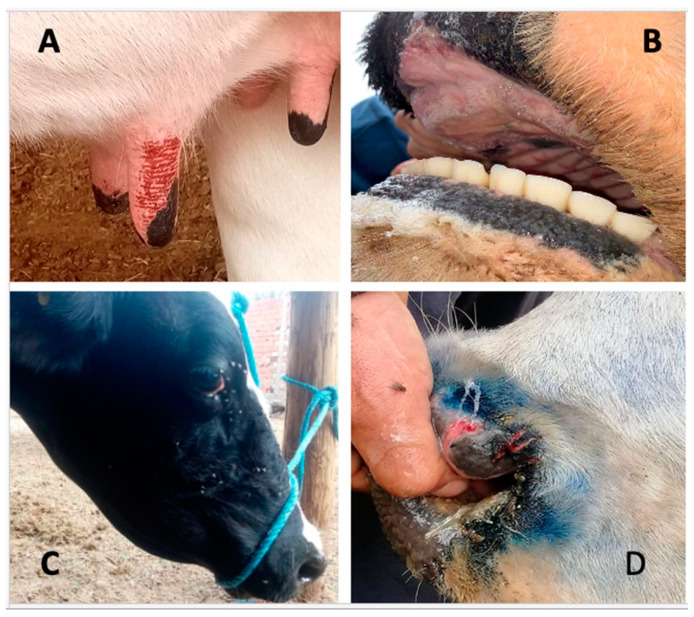
Clinical signs in cattle. (**A**) Teat erosions; (**B**) oral congestion and erosions; (**C**) submandibular oedema, conjunctivitis, and lacrimation; (**D**) nasal discharge and mucosal erosion. Reprinted/adapted with permission from the Tunisian Veterinary Services. Copyright 2021, copyright Dr Bel Hadj Mohamed Bassem (**A**,**C**); Dr Nadia Hemriti (**B**); Dr Meriem Ben Abdallah (**D**).

From late September to November 2021 a total of 174 whole blood and 241 serum samples were collected from cattle and delivered to the “Institut de la Recherche Vétérinaire de Tunisie” (IRVT), Tunisia. Cattle showed clinical signs including fever, conjunctivitis, lacrimation, drooling, erythema of nasal and oral mucosa and teat erosions (Figure 1). Most of the clinical cases were observed in late September 2021 in different delegations (the second level administrative divisions of Tunisia after the 24 governorates). The first outbreaks were reported in Central/Western Tunisia, and rapidly spread over a large part of the country with more than 200 outbreaks notified.

### 2.3. Serological Tests for EHDV

A total of 241 serum samples were screened for the presence of EHDV specific antibodies using a competitive ELISA (c-ELISA) test (Innovatives Diagnostics, Grabels, France). Of these, positive serum samples were tested using serum-neutralization (SN) tests against all reference EHDV serotypes, as described in the WOAH Manual.

### 2.4. EHDV Detection by Real Time RT-PCR and Genotyping

Total RNA was extracted from the blood samples using the QIAamp Viral RNA Kit (Qiagen, Hilden, Germany) and then tested for EHDV genome presence by real time RT-PCR using the VetMAX™ EHDV Kit (Thermo Scientific™, Waltham, MA, USA). This assay can detect a portion of Seg-9 of the known EHDV serotypes. EHDV-positive samples were further tested by means of genotype-specific assays targeting Seg-2 (encoding VP2) (rRT-PCR_VP2_) [22,23].

### 2.5. Virus Isolation and Virus Neutralization Test (VNT)

Sixteen whole blood samples with the lowest EHDV rRT-PCR cycle threshold (Ct) values were selected for virus isolation on BSR-T7/5-CVCL_RW96 (BSR) cells’ monolayers [24]. Five hundred microliters of EDTA blood were suspended in PBS and centrifuged at 800 rpm for 2 min. Red blood cells were then washed twice in PBS, resuspended in PBS and sonicated for approximately 20 s.

Lysed blood (0.2 mL) was diluted 1:5 *v*/*v* with MEM, then inoculated on pre-seeded BSR cells monolayer and incubated for 2 h at 37 °C. The inoculum was then replaced with MEM containing 10% foetal calf serum. After 5 days of incubation, the supernatant of the infected flask was collected separately, whereas the BSR cell monolayer was treated with trypsin to allow detachment. Once detached, the cells were suspended with 5 mL of the supernatant and the mixture transferred to a new pre-seeded BSR cell monolayer. After incubation and development of cytopathic effect (CPE) (approximately 5–7 days), cells were scraped off and EHDV replication was confirmed by rRT-PCR. Isolates positive for EHDV by rRT-PCR were tested [25] by VNT, in the presence of reference sera, for serological serotype characterisation. Four neutralizing units of reference sera (50 μL) were added to a 10-fold dilution series of EHDV (50 μL). The serum/virus mixtures were incubated for 1 h at 37 °C. BSR cells (20,000 cells in 100 μL/well) were added to each well and plates were incubated for 6 days at 37 °C with 5% CO_2_. A reduction of at least 2 logs is considered to classify a virus as belonging to the serotype of the antibody, which neutralized it.

### 2.6. Shotgun (SG) Metagenomic Analysis by Oxford Nanopore MinION

Three samples with different rRT-PCR Ct values (20–24–28) were selected for metagenomic shotgun (SG) analysis using the portable Oxford Nanopore MinION device to simulate a field-deployable whole genome sequencing scenario. Total RNA was treated with TURBO DNase (Thermo Fisher Scientific, Waltham, MA, USA) at 37 °C for 20 min and then purified by RNA Clean and Concentrator-5 Kit (Zymo Research, Irvine, CA, USA). RNA was then used for the assessment of sequence-independent single-primer amplification protocol (SISPA) with some modification [26]. The PCR products were purified by ExpinTM PCR SV (GeneAll Biotechnology Co., Seoul, Korea) and then quantified using the Qubit^®^ DNA HS Assay Kit (Thermo Fisher Scientific, Waltham, MA, USA). Library preparation was performed in approximately 20 min using the SISPA products and Rapid Barcoding Kit 96 (Oxford Nanopore, Littlemore, Oxford, UK) according to the manufacturers’ protocol. After flowcell priming, the library pool was loaded onto flowcell R9.4.1 (FLO-MIN106). The run parameters including the duration time (24 h) and basecaller model (Fast basecalling) were set-up, operating onto MinKNOW software. Fastq WIMP analysis was launched on EPI2ME platform to perform taxonomical classification of the fastq files in real time.

### 2.7. Illumina Genome Sequencing

To characterize the genome constellation of the novel EHDV, six whole blood samples with the lowest rRT-PCR Ct values were selected for Illumina genome sequencing. Total RNA was treated and purified, as previously described, and then used for the assessment of SISPA [26]. The PCR products were purified by ExpinTM PCR SV (GeneAll Biotechnology CO., Seoul, Korea) and then quantified using the Qubit DNA HS Assay Kit (Thermo Fisher Scientific, Waltham, MA, USA). Library preparation was performed using Illumina^®^ DNA Prep, (M) Tagmentation (96 Samples, IPB) (Illumina Inc., San Diego, CA, USA) according to the manufacturers’ protocol. Sequencing was performed on the NextSeq500 platform (Illumina Inc., San Diego, CA, USA) using the NextSeq 500/550 Mid Output Reagent Cartridge v2 (300 cycle) (Illumina Inc., San Diego, CA, USA) and standard 150 bp paired-end reads. After quality check and trimming of raw reads data using FastQC v0.11.5 and Trimmomatic v0.36, respectively, host depletion was performed by Bowtie2 [27]. The remaining reads were used for the de novo assembly using SPAdes 3.5 [28] and the consensus sequences of each genome segment were blasted against the NCBI database to identify related EHDV sequences.

### 2.8. Phylogenetic Analysis

EHDV nucleotide sequence data was obtained from NCBI (*n* = 1361 download 22 March 2022). The sequence data was separated according to genome segment. Sequences that were too short or had no sampling date were excluded. The final data set comprised: segment 1 *n* = 84 sequences, segment 2 *n* = 111, segment 3 *n* = 96, segment 4 *n* = 79, segment 5 *n* = 81, segment 6 *n* = 88, segment 7 *n* = 96, segment 8 *n* = 73, segment 9 *n* = 84, and segment 10 *n* = 73 sequences (total 866 sequences). A multiple sequence alignment was performed using the FFT-NS-2 algorithm in MAFFT [29,30] and manually inspected in Geneious Prime 2005–2022. Phylogenetic trees were estimated using the maximum likelihood method implemented in the IQ-TREE utilizing the best-fit model of nucleotide substitution and ultra-fast bootstrapping [31,32,33]. In the case of VP2 (segment 2) high levels of genetic divergence necessitated that phylogenetic trees were estimated using amino acid sequences. All phylogenetic trees were visualized in FigTree v1.4.4 (https://github.com/rambaut/figtree/releases accessed on 1 March 2022).

## 3. Results

### 3.1. Serological and Virological Tests Results

**Figure 2:**
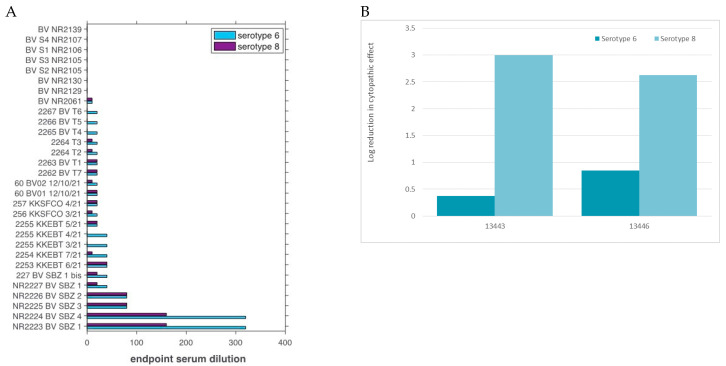
Virus neutralisation assays. (**A**) Serum neutralisation test results using EHDV serotype 6 (cyan) and serotype 8 (purple) reference isolates. (**B**) Values represent the log reduction in cytopathic effects (neutralisation).

Serum samples were only used for serological tests. Of 241 serum samples tested by c-ELISA, 160 were positive for EHDV antibodies. Of these, 30 samples had sufficient remaining material to also be tested by serum-neutralization for serotype-specific EHDV antibodies. The 30 EHDV positive sera showed a neutralization only for EHDV-6 (EHDV-6 AUS1981/07) and EHDV-8 (AUS1982/06) reference isolates but not for the other EHDV serotypes tested. Five of 30 samples were positive for EHDV-6-specific Abs only, and an additional 18/30 were positive for both EHDV-6 and EHDV-8 specific Abs (Figure 2A). The remaining 7 sera were negative for either serotype likely due to lower sensitivity of the neutralizing antibody assay at early stages of infection. No significant difference was found in the titres of the sera from cattle obtained with the two reference strains EHDV-6 and 8, suggesting serological homogeneity. Of 174 whole blood samples analysed, 55 were positive for EHDV by rRT-PCR. However, the EHDV genotype-specific rRT-PCR_VP2_ failed on all 55 EHDV-positive samples.

### 3.2. Virus Isolation and VNT

Virus isolation was successful only for five samples, and the presence of EHDV in cell culture was confirmed by rRT-PCR. Two isolates (#13443 and #13446) were used in VNT against EHDV serotype 6 and 8 reference antiserum. Both samples showed a 2-log or higher reduction in neutralisation against serotype 8 reference antiserum, but only a 0.37 and 0.85-log reduction in neutralisation against serotype 6 reference antiserum (Figure 2B).

### 3.3. EHDV Genome Identified by Shotgun Metagenomic Sequencing

Three EHDV samples with Ct values of 20, 24, and 28 were processed for shotgun metagenomic sequencing. Detectable sequence reads classified as EHDV were produced within 20 min from the beginning of the run. At the end of the run (after 24 h), the number of reads classified as EHDV per sample was 47,060 (C_T_ value 20), 23,555 (C_T_ value 24), and 1980 (C_T_ value 28) with an average quality score of 10.64. Unfortunately, it was not possible to genetically characterise the EHDV serotype as the database related to the What’s in my Pot? (WIMP) application does not include EHDV reference sequences for all EHDV serotypes.

### 3.4. The Whole Genome Sequence of EHDV-8/17 TUN 2021 Strain

Six samples with the lowest Ct values were selected for whole genome sequencing using the Illumina MiSeq technology. The total number of raw reads produced from each sample varied from 1717,478 to 3078,122 with average length of 143 bp. The *de novo* assembly produced nearly complete consensus sequences for each genome segment for all six samples. Notably, all sequences obtained showed >99.9% nt identity to each other (excluding missing regions due to sequencing failure). Isolate EHDV-8/17 TUN2021 had the best overall horizontal and vertical coverage and was therefore considered representative and hence deposited in GenBank (acc. nos OP381190-OP381199).

A *blastn* and *blastp* search was performed for all ten segments of EHDV-8 TUN 2021. For segment 2, only one match was found with *blastn* with only 76.53% identity to a serotype 8 isolate from Australia 1982 (Table 1). This is the only other serotype 8 isolate ever reported. Three segments (5, 7, and 8) where closest to a South Africa EHDV-6 isolate from 1996, with ~98% identity. Segments 1 and 9 were genetically close to a serotype 7 isolate from Israel 2006 (~98% identity), with segments 4 and 10 closest to a serotype 1 isolate from Nigeria 1968 and 1967 (93% and 95% identity, respectively). Finally, segment 3 was closest to a Tunisian serotype 6 isolate from 2006, while segment 6 was closest to the reference serotype 8 from Australia, but with only 80.52% identity.

**Figure 3:**
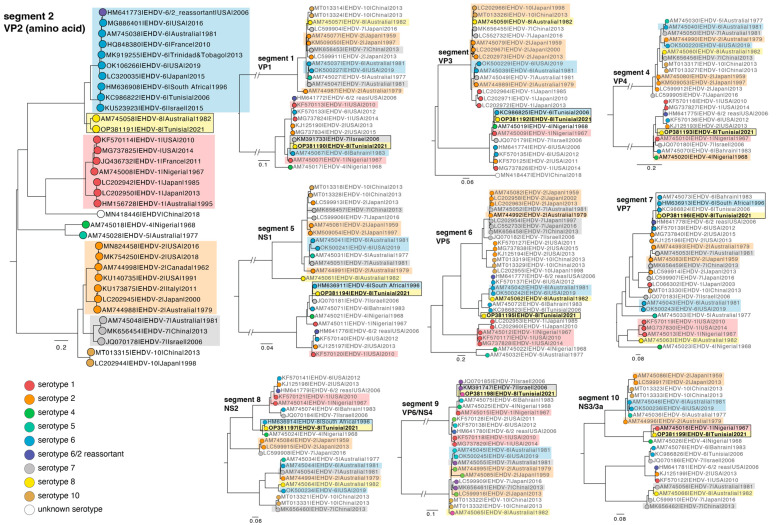
Phylogenetic trees for EHDV segments (amino acid sequences for segment 2, nucleotide sequences for all other segments). Maximum likelihood trees were estimated using IQ-TREE employing the best-fit model of substitution. The trees shown are a representative selection of the different serotypes to simplify visualisation. The different serotypes are indicated. Trees are annotated according to serotype (segment 2) and sequences from the study here are shown in bold and yellow box (Tunisia 2021). *Blastn* outputs are shown in black. Branch length indicates the number of nucleotide substitutions per site for segments 1, 3–10 and amino acid substitutions per site for segment 2. Note, a putative new serotype has only recently been reported in viruses from China (white) [10]. Number of sequences used in the displayed trees; segment 2 *n* = 34, segment 1 *n* = 24, segment 3 *n* = 25, segment 4 *n* = 23, segment 5 *n* = 23, segment 6 *n* = 30, segment 7 *n* = 19, segment 8 *n* = 22, segment 9 *n* = 22, and segment 10 *n* = 21 sequences.

The sequences obtained from the *blastn* output and other publicly available sequence data for EHDV were combined with the sequences obtained in the present study. Segment 2, that encodes VP2, was used to assign the virus sequences into different serotypes (Figure 3). Accordingly, the segment 2 phylogeny placed the sequences obtained in the study here as closest to the serotype 8 sequence from Australia isolated in 1982, consistent with the *blastn* results (Figure 3). Indeed, the phylogenetic analysis corroborated the *blastn* results for most segments. The Tunisian isolate segment 1 (VP1) and 9 (VP6/NS4) formed a monophyletic group with serotype 7 isolates from Israel 2006. Segment 3 was closest to the serotype 6 isolate from Tunisia from 2006, and segments 5 (NS1) and 7 (VP7) were closest to serotype 6 isolates, including South Africa 1996). The segment 10 (NS3/3a) sequence fell closest to serotype 1 virus from Nigeria 1967. Notably, the Tunisian isolate segment 8 (NS2) formed a monophyletic group with the South Africa serotype 6 isolate as per *blastn* output, but also with the serotype 4 isolate from Nigeria 1948. Interestingly, the segment 4 (VP4) phylogeny showed that the Tunisian isolate grouped with serotypes 2, 6, 1, while the isolate serotype 4 from Nigeria identified in the *blastn* analysis fell basal to this group. For segment 6, the Tunisian and the Australian serotype 8 isolates were sister clades and formed a monophyletic group with serotype 6 isolates.

## 4. Discussion

We describe the identification of EHDV serotype 8 causing outbreaks in cattle in Tunisia. In addition to the threat posed to Tunisian livestock, the geographic proximity to Europe represents a clear risk for its even wider spread. Notably, EHDV and BTV occupy a similar biological niche and the latter spreads repetitively to Europe via *Culicoides* in sandstorms. Surveillance is therefore crucial to mitigate disease spread across the continents.

Our initial serological investigations (ELISA and serum neutralization) performed on serum samples allowed for the accurate identification of the serotype. Serologically, the difference between the titres obtained against the two reference EHDV-6 and 8 strains was not significant, such that initial diagnosis was made based upon the most realistic scenario, with EHDV-6 as the serotype in question, as this has been circulating in Tunisia previously (2006). In addition, serotype 8 virus had only been documented once before in Australia in 1982. However, our genome sequencing analysis revealed that the Tunisian strain identified here was closest to this Australian isolate and the virus neutralization results also demonstrated that the EHDV Tunisian strain belongs clearly to the EHDV serotype 8.

In combination, our phylogenetic results suggest that EHDV-8/17 TUN2021 has a genome constellation composed of gene segments that are genetically close to homologous segments of EHDV strains identified in the African continent (segments 1, 3, 4, 5, 7, 8, 9, and 10), with the exception of segments 2 and 6 that were more closely related to the only EHDV-8 strain ever isolated from Australia in 1982. Importantly, however, this does not mean that segments 2 and 6 of the Tunisian strain have an Australian origin, as the geographic sampling of these viruses is so limited, with only a few available sequences available for most years. Indeed, it is striking that the most recent sequences deposited on NCBI prior to 2021 are isolates from Florida from 2019, sampled during an outbreak in white-tail deer [34]. In addition, only one other sequence was available from Tunisia, serotype 6 from 2006, and only four sequences were available from other African countries.

Another notable result was that the currently available molecular PCR-based typing methods failed at accurate EHDV serotyping, most likely due to the genetic variation in the VP2 nucleotide sequence between EHDV-8/17 TUN2021 and the EHDV-8 Australia 1982 isolate used as reference for serotype 8 specific assays. In addition, using a portable MinION device for ‘on-site’ shotgun sequencing and characterisation also failed to classify the serotype accurately due to a lack of appropriate reference sequences. We hope that our study will aid in updating these methods, as the Oxford Nanopore MinION technology would greatly enhance the early detection, notification, and assessment of containment measures for future outbreaks.

Currently, the only valid strategy for EHD prevention is vaccination. Unfortunately, vaccine development for EHDV remains poor. The reported outbreaks of EHDV-8 in Tunisia, and the potential co-circulation of multiple serotypes, highlight the need for prompt production and release of new vaccines. As this EHDV-8 strain has been shown to induce significant clinical signs, including mortality, in infected cattle. Thus, we strongly believe that countermeasures must be taken. These include the establishment of EHDV surveillance in the European regions previously shown to be at high risk of *Orbivirus* incursions from Northern Africa [35,36,37,38], entomological studies to assess the biological vector of EHDV-8 in Tunisia, surveillance of the circulation of this virus in other animal species including camelids [39], and the production of specific EHDV-8 vaccines able to protect animals from clinical disease. In this regard, it should be noted that a vaccine designed on EHDV-6 could be also effective at preventing EHDV-8 clinical disease, but this must be proven in dedicated experimental in vivo trials.

In sum, we present a new EHDV-8 isolated from an outbreak in Tunisia, which is only the second strain characterized belonging to this serotype. The neutralization results confirm that this EHDV-8 strain is close to serotype 6 and belongs to the nucleotype B, as previously described [7].

## Figures and Tables

**Table 1:** Blast results for nucleotide and amino acid sequences for EHDV-8 /17 TUN 2021 strain.

Accession Nr	Segment	Blastn Output Strain (Accession Number)	% Identity
OP381190	1	Israel 2006 ISR2006/04 serotype 7 (KM391733)	98.58
OP381191	2	Australia 1982 CPR_3961A serotype 8 (AM745058)	76.53
OP381192	3	Tunisia 2006 2577 serotype 6 (KC986825)	97.41
OP381193	4	Nigeria 1968 IbAr33583 serotype 4 (AM745020)	93.29
OP381194	5	South Africa 1996 M44/96 serotype 6 (HM636911)	97.55
OP381195	6	Australia 1982 CPR_3961A serotype 8 (AM745062)	80.52
OP381196	7	South Africa 1996 M44/96 serotype 6 (HM636913)	98.01
OP381197	8	South Africa 1996 M44/96 serotype 6 (HM636914)	98.31
OP381198	9	Israel 2006 ISR2006/04 serotype 7 (KM391738)	97.63
OP381199	10	Nigeria 1967 IbAr22619 serotype 1 (AM745016)	95.79

## Data Availability

The data presented in this study are available upon request to the corresponding author. Nucleotide sequences of viral strains are available on GISAID.
